# Radiolabeling of *Angiostrongylus vasorum*- and *Crenosoma striatum* larvae: a novel method using PET/CT to unveil larval migration in the gastropod intermediate host (*Lissachatina fulica*)

**DOI:** 10.1186/s13071-025-07088-0

**Published:** 2025-11-18

**Authors:** Joep T. van de Sanden, Alena Dusch, Katharina M. Westhoff, Monique R. Bernsen, Anja Taubert, Carlos Hermosilla, Frederik A. Verburg

**Affiliations:** 1 Department of Radiology and Nuclear Medicine, Erasmus Medical Centre, Rotterdam, The Netherlands; 2 Institute of Parasitology, Justus Liebig University Giessen, Giessen, Germany; 3 AMIE Core Facility, Erasmus Medical Centre, Rotterdam, The Netherlands; 4 Department of Nuclear Medicine and Core Facility Small Animal Imaging, Philipps-University Marburg, Marburg, Germany; 5 Cyclotron Rotterdam B.V., Rotterdam, The Netherlands

**Keywords:** *Lissachatina fulica*, Radiolabeling, *Angiostrongylus vasorum*, *Crenosoma striatum*, Gastropod-borne diseases

## Abstract

**Background:**

Gastropod-borne metastrongyloid lungworm infections are poorly understood despite their importance in both veterinary and human medicine. This study intends to assess the use of nuclear imaging with whole-body positron emission tomography and computed tomography to scan radiolabeled *Angiostrongylus vasorum*- and *Crenosoma striatum* first-stage larvae (L1) while migrating in vivo in the obligate mollusc intermediate hosts. Here, the giant African snail (*Lissachatina fulica*) was used as a novel animal model for lungworm-associated investigations, as this gastropod species is known to act as a natural obligate intermediate host in the tropics for various metastrongyloid lungworms, including the zoonotic-relevant *Angiostrongylus cantonensis*. Radiolabeled *A. vasorum*- and *C. striatum* L1 migration was visualized through nuclear imaging after L1 oral infection or injection.

**Methods:**

Live L1 were collected through the standard Baermann funnel technique. After isolation and assessment of larval viability, collected *A. vasorum*- and *C. striatum* L1 were incubated with a radiolabeled glucose analogue, ^18^F-fluordesoxyglucose. Thereafter, radiolabeled L1 were fed orally or injected into adult *L. fulica*, and in vivo scans were performed to visualize larval migration routes at different time points post infection through various gastropod organs/tissues.

**Results:**

The most optimal incubation time of larvae and ^18^F-fluordesoxyglucose was 30 min. After washing nonincorporated tracer, metastrongyloid L1 retained on average activity of 0.33 (0.103) KBq per larvae. This was the maximum activity achieved, and even longer incubation times, i.e., 60 and 120 min, did not exceed this value. The in vivo scans showed dispersal from the site of larval injection or feeding. Radiolabeled *A. vasorum*- or *C. striatum* L1 moved rapidly from the site of injection or the oral cavity with nonspecific accumulation in one or numerous gastropod organs at 30 min post infection.

**Conclusions:**

This study concludes that radiolabeling of metastrongyloid L1 with ^18^F-fluordesoxyglucose is achievable up to a level that can be detected in a scan of individual snails. Scanning L1 larvae in *L. fulica* at 60 min still represents larval migration. After 2 h, this study found that the migration is already widespread, and the activity is too low to be narrowed to a specific organ. Detailed in vivo scans of gastropods with not only higher infectious doses but also other radiolabeled tracers and longer observation periods might allow detection of either organ tropism or larval accumulation in certain mollusc tissues/organs for further development into infective stages.

**Graphical abstract:**

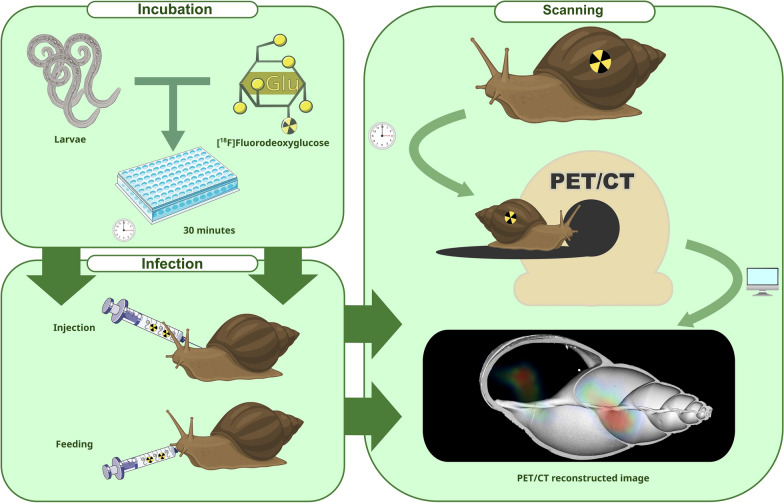

**Supplementary Information:**

The online version contains supplementary material available at 10.1186/s13071-025-07088-0.

## Background

Gastropod-borne parasitic diseases, such as metastrongyloid lungworm infections of humans and animals, are rarely addressed in research. In Europe, multiple species of metastrongyloid lungworm species infect various definitive hosts, both domestic and wild animals. Two highly prevalent lungworms in Europe are *Angiostrongylus vasorum* and the closely related *Crenosoma striatum*. *A. vasorum* infects domestic dogs, wild canids (e.g., wolves, foxes, and jackals), badgers, raccoons, and red pandas [1–5]. All these carnivore species act as definitive hosts (DH) where the adult nematodes reside in the right heart and in the pulmonary artery. Canine angiostrongylosis may lead to a variety of symptoms, ranging from mild to severe ones. Clinical signs include respiratory symptoms, hemorrhages, coagulopathies, neurological and ophthalmic disorders, and gastrointestinal symptoms [5, 6].

Conversely*, C. striatum* is commonly found in the lungs of European hedgehogs (*Erinaceus europaeus*). Respiratory symptoms of crenosomosis in affected hedgehogs include dry cough, bronchitis, hyperthermia, and weight loss [7, 8].

The life cycle of these lungworms is heteroxenous, with terrestrial gastropods (i.e., snails, semi-slugs, and slugs) acting as obligatory intermediate hosts (IH) [9, 10]. Terrestrial gastropods become infected by feeding either on the feces containing first-stage larvae (L1) from an *A. vasorum*-infected DH or from a *C. striatum*-infected European hedgehog. While inside the gastropod IH, the larvae develop into the second-stage larvae (L2) and third-stage larvae (L3), which are then infective for other DH. Therefore, metastrongyloid infections of canids include direct gastropod consumption carrying L3, consumption of paratenic hosts (PH) such as mice, reptiles, and frogs feeding on infected IH, and/or infective L3 in the environment released from either dead IH or PH, for example, in grass or water [11].

The giant African snail *Lissachatina fulica* is one of the largest terrestrial snails in the world. Originally endemic to East Africa, this gastropod species is currently regarded as one of the most invasive gastropod species worldwide. Stable populations of this mollusc neozoon were found among others in Ecuador, the USA, Brazil, India, and Colombia [12–14]. Moreover, *L. fulica* is known to be an important IH for multiple parasite species, including various lungworm species [13, 15].

There is still little knowledge about the development of metastrongyloid lungworm larvae, organ tropism, and host innate immune reactions against these stages inside the gastropod [16, 17]. To obtain precise data on the parasite’s location and distribution speed inside terrestrial gastropods in vivo, imaging techniques can be used. Nuclear imaging with whole-body positron emission tomography and computed tomography (PET/CT) is a well-established technique in the medical field to study various physiological processes within the body, for example, to image and assess tumor sizes and even progression [16]. This noninvasive/minimally invasive technique offers a unique opportunity to observe precise processes in vivo*.*

For example, ^18^F-fluordesoxyglucose (^18^F-FDG) is a radiolabeled tracer that is being used in PET/CT scanning. It is widely available and routinely employed in clinical practice. With a physical half-life of approximately 109 min, it is classified as a short-lived radionuclide. While clinical protocols typically utilize activity around 1 GBq, a substantially lower activity of 10 MBq was selected for this study to accommodate the smaller body size of snails, consistent with dosing regimens commonly applied in preclinical studies involving mice and rats. As a glucose analogue, ^18^F-FDG is considered nontoxic at concentrations that do not exceed physiological levels; it is taken up in larger amounts by cells with high metabolic activity, such as in tumors, the brain, the liver, and even parasites [18, 19]. Nematodes, especially, are known to consume and metabolize glucose; as such, the use of a radioactive glucose derivative (^18^F-FDG) is a suitable approach for tracking larval migration [20, 21].

In our study, we used nuclear imaging to visualize the distribution over time of relevant metastrongyloid parasite species within *L. fulica*, which can be infected with ease under experimental conditions. The overall aim of this study is to assess the feasibility of radiolabeling *A. vasorum*- and *C. striatum* larvae to visualize their endogenous migration within this large terrestrial gastropod, with the intention of determining their precise route of migration and identification of organ tropism. Understanding the localization of *A. vasorum*/*C. striatum* larvae or other lungworms might lead to not only a better understanding of parasite–*L. fulica* interactions but also to hampering transmission to various mammalian DH, including humans, as is the case for zoonotically relevant *Angiostrongylus cantonensis* and *Angiostrongylus costaricensis*, both species circulating in *L. fulica* populations in the tropics.

## Methods

### Parasite isolation

*Angiostrongylus vasorum*- and *C. striatum* L1 were acquired by isolation from naturally infected dogs and European hedgehogs, respectively. Fresh feces were collected and thereafter examined via the Baermann–Wetzel apparatus according to Conboy et al. [22]. The hedgehog feces were kindly provided by the *Egelopvang Papendrecht* (the Netherlands) and the *Wildtierfreunde e.V.* (Germany). The dog feces originated from the Parasitology Diagnostic Unit (PDU) of the Institute of Parasitology, Justus Liebig University Giessen, Giessen, Germany.

Collected larvae were stored in small tubes at 4 °C until used for snail infections. To achieve a higher larval concentration, the solution containing the larvae was centrifuged for 5 min at 800*g* at room temperature (RT). The supernatant was discarded, and the larvae pellets were resuspended in physiological saline after concentration. Using this method, an infectious dose (ID) of either 1000 or 2000 L1 per snail was created by counting the larvae under a light microscope.

### Larval radiolabeling in vitro

^18^F-FDG was procured from the Nuclear Department, Faculty of Human Medicine at the Philipps-Universität Marburg, Marburg, Germany. Approximately 1500 *A. vasorum* larvae were divided into five groups: three live study groups (A, B, C) incubated with 1 MBq of ^18^F-FDG per well and two negative control groups (DF and LN) (Table 1). Each of the five groups was further divided into six subgroups varying in incubation time (5, 10, 15, 30, 60, and 120 min). Every subgroup was pipetted in an individual well of a 96-well plate (Greiner, Sigma-Aldrich, Germany). For each incubation time point, every well corresponding to a subgroup contained approximately 50 larvae. After incubation, each group was washed three times with phosphate-buffered saline (PBS) by centrifuging them at 800*g* (RT) for 5 min and resuspending the specimens in PBS.

**Table 1:** Radioactive uptake in a single *Angiostrongylus vasorum* larva, calculated for live larvae and dead larvae at each time point

Minutes	A + B + C in kBq/larva (SD)	Dead larvae with ^18^F-FDG control in kBq/larva
5	0.08 (0.013)	0.04
10	0.07 (0.016)	0.02
15	0.11 (0.067)	0.04
30	0.33 (0.103)	0.1
60	0.22 (0.084)	0.21
120	0.3 (0.128)	0.04

The number of larvae in a well was divided by the kBq value for each well corresponding to a time point and group. A, B, and C represent three groups of live larvae that were incubated with ^18^F-FDG for a specified number of minutes. The result at each time point for A, B, and C was averaged into a single activity per larva. Dead larvae with ^18^F-FDG control consist of approximately 50 dead larvae per well per time point that were incubated with ^18^F-FDG for a specified number of minutes. As there was only one dead larvae control group, the results are not averaged

*SD* standard deviation

The DF and LF groups served as negative controls to validate our results. The DF group consisted of dead larvae that were also incubated with 1 MBq of ^18^F-FDG. In contrast, the LF group consisted of living larvae with the same washing steps as the positive groups, but without an ^18^F-FDG incubation.

Finally, the larvae were moved to counting tubes and were loaded onto a Perkin Elmer^®^ gamma counter. The activity per larva was calculated by dividing the readings from the gamma counter by the total number of larvae that were counted under the microscopes. To determine the activity linked to a reading and to correct for ^18^F-FDG decay, two reference samples, vials containing only ^18^F-FDG with a known activity of 25 kBq and 50 kBq, were loaded into the gamma counter. The readings from these two samples were used to approximate the amount of activity linked to the readings for each larval sample.

### Larval labeling for imaging

The ^18^F-FDG used in imaging was obtained as stated above from the Nuclear Department at the Philipps-Universität Marburg (Germany). For the second set of scans, performed in Rotterdam, the ^18^F-FDG was produced by Cyclotron Rotterdam B.V., the Netherlands. The larvae were retrieved from the fridge in concentrated form, assessed for viability and ID, and brought to the radioactive workbench. The L1 were combined with ^18^F-FDG activity at 30 MBq and incubated at RT for 30 min. The larvae were then washed three times in PBS by centrifuging them for 5 min at 800*g* on an Eppendorf cup centrifuge and by pipetting off the supernatant and resuspending it in H_2_O. Finally, the activity was measured and noted down as previously described.

### Snails

Ten giant African snails (*L. fulica*) were held in a climate chamber (ECP01E, Snijders Scientific B.V., Tilburg, the Netherlands) at the Institute of Parasitology, JLU Giessen, Giessen, Germany. The light cycle consisted of 10 h of light and 10 h of darkness with 2 h for dawn and dusk, respectively. The snails were fed ad libitum with cucumbers, zucchinis, carrots, green lettuces, and commercial dog food as reported by Dusch et al. (2024). As a natural calcium source, shells of the cuttlefish (*Sepia* spp.) were used here.

### Snail infection with either radiolabeled ^18^F-FDG *A. vasorum*- or *C. striatum* L1

The snails were carefully cleaned with running water before larval infection and thereafter placed inside a radioactive workbench. The radiolabeled ^18^F-FDG L1 of *A. vasorum* suspended in solution were injected near the pneumostome as described by Cooper (1995), placing them close to the gastropod central hemolymph circulation system promoting a quick distribution. After this procedure, the snails were returned to their boxes to wait for 5 min for larval migration. Before PET/CT analysis, the snails were immobilized using an ice bath and placed on the bed for the scanning machine (NanoScan, Mediso).

For the second set of scans, performed in Rotterdam, we decided against parasite injection and opted for voluntary oral infection instead, to be as close as possible to the in vivo situation where larvae (i.e., L1) are ingested by coprophagic gastropods. The snails were fed the radiolabeled ^18^F-FDG L1 of *C. striatum* suspended in physiological saline solution with a needleless syringe orally. The rest of the scan protocol was the same as for the metastrongyloid-injected snails.

### PET/CT imaging

PET/CT images (NanoScan, Mediso) were acquired in six with *A. vasorum*-infected *L. fulica* (*n* = 6) at 30, 60, and 90 min post infection (p.i.). A seventh non-infected snail (*n* = 1) was injected only with ^18^F-FDG to serve as a control and scanned afterward in the same way as the infected snails. The CT was obtained using the following parameters: 70 kV, 84 µAs, and one projection. Positron emission tomography (PET) scans were performed using a whole-body window. The first scan was a dynamic scan consisting of ten short scans at a 1-min interval. Images were reconstructed using a reconstruction program and the MLEM algorithm [23].

For the second set of scans, performed in Rotterdam, PET/CT images (VECTor/CT, MILabs, the Netherlands) were acquired in three with *C. striatum*-infected *L. fulica* (*n* = 3). All three of the snails were scanned at 5 min p.i., and two of these snails at 2 h p.i. as well. The CT was obtained using the following parameters: 55 kV, 0.17 μA, and one projection. PET scans were performed using the ultrahigh-definition mouse–rat collimator, scanning four time-windows of 5 min. Images were reconstructed using the MILabs^®^ reconstruction program using the SROSEM method with the following parameters: voxel size 0.8 mm, 50 iterations, and 16 subsets. For segmentation and post analysis, Imalytics^®^ preclinical software (Gremse-IT, Germany) was used.

### Statistics

For statistical analyses, RStudio [2024.09.0 Build 375(R-4.4.2)] was used. The data were separated into three groups, and a linear regression was performed for the time points 5–30 min and the time points 30–120 min to determine if there was a relation between minutes and uptake per larva. Finally, the averages for the different groups were calculated at each time point. All data are displayed as follows: mean (standard deviation [SD]). Significant values of *P* < 0.05 were considered.

## Results

### Larval incubation

Incubation of live *A. vasorum* L1 with ^18^F-FDG resulted in specific uptake in the larvae, which increased between 5 and 30 min after incubation. The ^18^F-FDG uptake per larva increased from 0.08 (SD = 0.0126) kBq to 0.33 (SD = 0.103) kBq per larva (Table 1). A simple linear regression model demonstrated that the uptake time explained a significant amount of the increase in activity per larva for the time points 5–30 min (*t*_(5)_ = 5.519,* P* < 0.001). In contrast, in the linear regression model for time points 30–120 min, respectively, uptake time was not a predictor for activity per larvae (*t*_(5)_ = −0.12, *P* = 0.909). After 30 min of exposure, for the time points 30, 60, and 120 min combined and averaged, the activity leveled out at 0.282 (SD = 0.080) kBq per larva.

### PET/CT scans

**Fig. 1:**
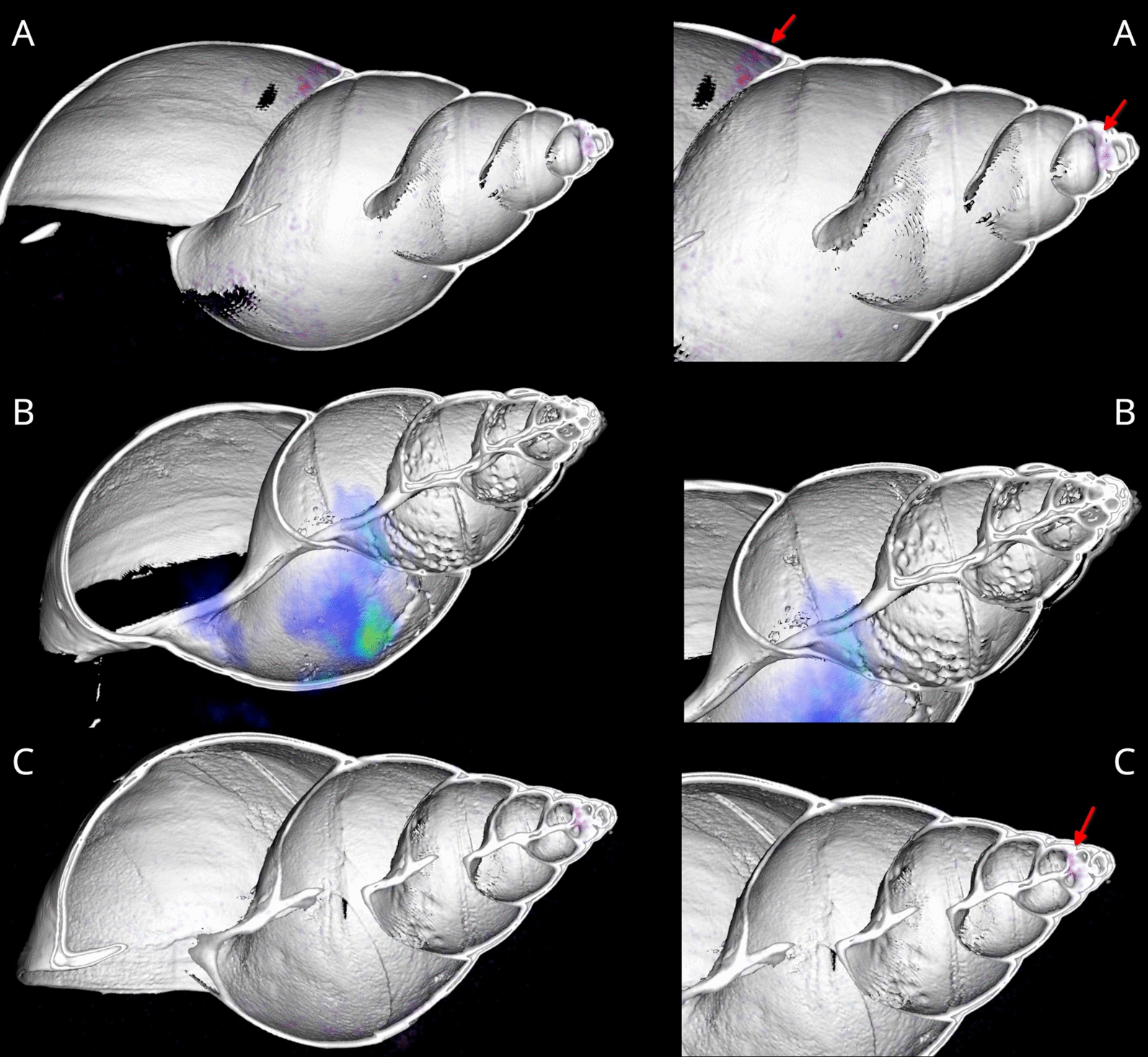
PET/CT image of two *Lissachatina fulica* snails (**A**, **C**) and one control (**B**) scanned 60 min after injecting ^18^F-FDG-radiolabeled *Angiostrongylus vasorum* larvae. **A**, top; red arrows: activity in the digestive tract and the digestive gland. **B**, middle: control. **C**, bottom: digestive gland visible

PET/CT images revealed hotspots immediately after injection, primarily in the snail’s mass dorsally from the pneumostome. These indicated limited dispersal in 30 min. Conversely, 60 and 90 min after injection, the scans using *A. vasorum* L1 displayed a more widespread larval distribution throughout the gastropod’s body, which is indicative of advanced larval migration (Fig. 1). The activity, however, was in the lower digestive tract and the albumen gland. Two snails showed a distinct distribution that was not found in the control ^18^F-FDG-injected snail, a focal point in the top part of the digestive gland (Fig. 1).

**Fig. 2:**
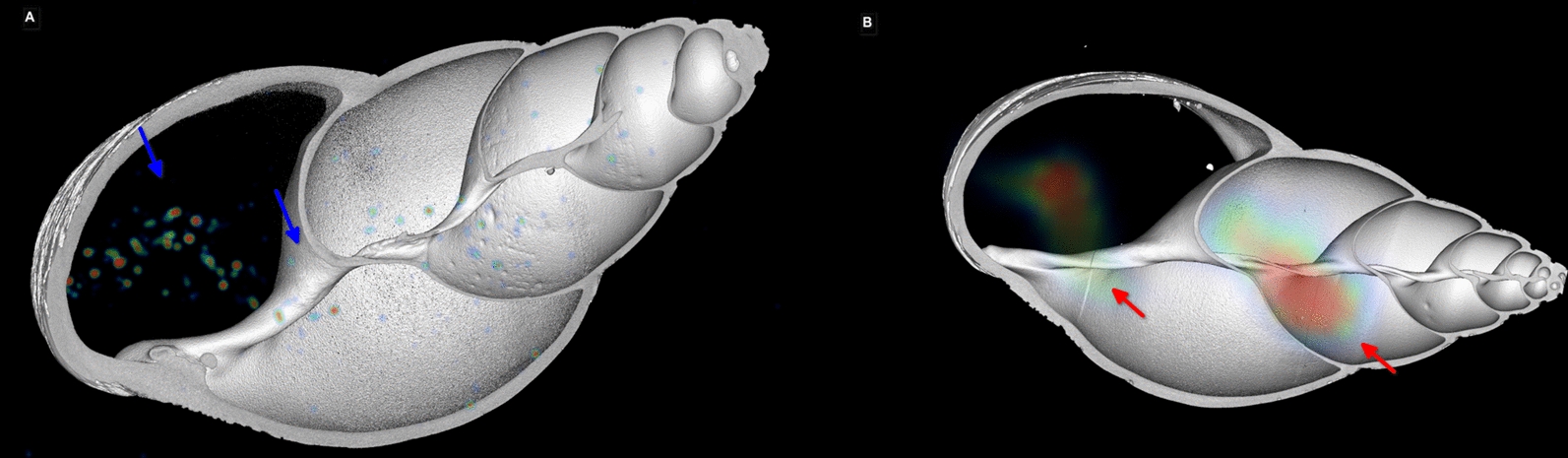
PET/CT image of two *Lissachatina fulica* snails scanned 10 min after orally feeding ^18^F-FDG-radiolabeled *Crenosoma striatum* larvae. **A**, Snail, top-view uptake in digestive tract up to the stomach, blue arrows. **B**, snail 2, top-view uptake in the digestive tract, red arrows

In the orally infected snails, *C. striatum* larvae uptake was observed primarily in the snail’s digestive tract. These indicated larval dispersal from the esophagus, the crop, and, in one subject, into the stomach (Fig. 2). After 2 h of larval post-feeding, the scan displayed a more widespread distribution throughout the gastropod’s body, which is indicative of advanced larval migration. The ^18^F-FDG activity, however, was too low to narrow this uptake to a specific inner organ. As such, precise scans could only be performed up to 2 h after injection.

## Discussion

This study set out to test whether it was possible to incubate metastrongyloid lungworm larvae with ^18^F-FDG and to scan them thereafter in vivo in giant African snails (*L. fulica*), which are known to act as suitable obligate IH of various metastrongyloid lungworm species in the tropics of Colombia [13] and Brazil [24].

We decided to use two different lungworm species owing to their differences in localization in the IH. As such, the larvae are exposed to different stressors, which may result in different reactions to both the experimental conditions as well as the organ tropism of the larvae.

The first findings of this work reveal that live metastrongyloid L1 of *A. vasorum* and *C. striatum* can successfully be exposed to the ^18^F-FDG tracer and after ingestion achieving sufficient radioactivity levels for effective PET/CT scanning. The uptake of ^18^F-FDG in *A. vasorum* L1 larvae reached approximately 0.3 KBq after 30 min and did not increase any more. This could indicate that the larvae were satiated or that the maximum amount of ^18^F-FDG was ingested. However, the various replicates showed a difference in the average activity per larva. A possible explanation for this could be individual variance in the isolated larvae, for example, due to larval size and/or metabolic activities or individual larval stress.

The scans performed after injection showed larval activity arising from the injection spot as well as from the lower part of the gastrointestinal tract in four individuals (*n* = 4). These results seem to be consistent with other research, which found that the injection method used resulted in most parasites remaining close to their insertion point into the snail, thereby causing a higher number of larvae to be collected from these regions [25–27].

The remaining scans that were performed every 30 min after injection demonstrated a widespread pattern that could not be linked to one specific organ. It is therefore likely that the larvae migrated toward different organs, thereby leaving the gastrointestinal tract. A study by Sauerländer et al. (1976), who used infection doses of 5000 and 20,000 larvae per snail, found that, within 2 h, a large quantity of *A. vasorum*- and *A. cantonensis* larvae had moved toward the lung and foot of the snails [15, 25, 28–30]. Surprisingly, we did not find such a specific signal coming from either organ on the scan after the follow-up period of 2 h. This might be explained by the large volume of both organs and the possibility that the signal was diluted by the lower infection dose the snails received in our study. A distinct signal was however observed at the top of the shell in the digestive gland for two of the snails in the “larval-injected” scans.

In an attempt to mimic a natural infection route and to achieve more accumulation of parasites, the second snail scans occurred after an oral infection route for these parasites. Two different metastrongyloid lungworm species (*A. vasorum* and *C. striatum*) were used to show possible species disparities in migration. In these “larval-oral” scans, the digestive tract of animals showed a large amount of uptake right after initiation of PET/CT scans. Nonetheless, with subsequent scans, the amount of uptake visible in the digestive tract decreased and after 2 h was completely absent. Interestingly, there was no spot visible in the digestive gland.

The most important limitation of radiolabeling lies in the fact that even in the DF control group, a small amount of ^18^F-FDG uptake is observed, which suggests that our washing protocol was not sufficient to completely wash away all the remaining ^18^F-FDG. Because of the small sample size (*n* = 7), caution must be applied, and, in further studies, the effect of this phenomenon should be minimized by increasing the number of replicates and by improving larval washings. This observation was also present in the first scans performed after larval injection. A large amount of activity was observed from the injection site; however, contamination with the remaining ^18^F-FDG, which could not be completely washed out of the injection solution, could be a contributing factor to the activity present in this region. Finally, the scans showed some overlap with the control scan, which also unveiled a lot of activity coming from the digestive tract and albumen gland (Fig. 1).

Considering the half-life of ^18^F-FDG (109.8 min), the signal is halved approximately every 2 h. This by itself could have contributed to the diluted signal found in all infected scans. For a follow-up, the usage of longer half-life radionuclides or a higher initial activity could be more precise in following the larvae during migration to allow scanning over a longer time period. A tracer that is used for nuclear cell imaging, like ^111^Indium-oxine, could be an option as it is used for immune-cell tracking [31].

This study set out to establish whether it was possible to radiolabel metastrongyloid lungworm larvae and track larval migration in vivo within gastropods via a PET scan. The most obvious finding to emerge is that it was feasible to label the parasitic larvae with ^18^F-FDG after 30 min of incubation and that these parasitic stages could be visualized in an early state of their endogenous gastropod migration with a PET/CT scan.

## Conclusions

In this study, we were able to show that ^18^F-FDG-radiolabeling of vital L1 of two endemically occurring metastrongyloid species in Europe, that is, *A. vasorum* and *C. striatum*, can be achieved to track migration routes within gastropods with minimally invasive (injection of larvae) and with noninvasive (oral uptake of larvae) methods. Despite the low number of snails used here, the study certainly adds knowledge to better our understanding of metastrongyloid parasite migration and localization within mollusc IH. The scans could not pick up a signal after 2 h p.i. that was clear enough to narrow the activity to organs or migration paths. However, this initial work should be repeated and tested on a larger sample of gastropods, with higher infectious doses and with other radiolabeled tracers. Thus, different radionuclides as well as other lungworm species, including zoonotic ones (*A. cantonensis* and *A. costaricensis*) can also be considered in the future since *L. fulica* is known to be a suitable IH in the tropics. The same holds true for species of veterinary relevance in felids and canids such as *Troglostrongylus brevior*, *Aelurostrongylus abstrusus*, and *Crenosoma vulpis*.

## Supplementary Information

Supplementary Material 1. Fig. S1 pictures of first stage larvae of *Crenosoma striatum* and *Angiostrongylus vasorum*

Supplementary Material 2. Table S1 raw readings

## Data Availability

All data generated or analyzed during this study are included in the article and its additional files.
