# Supplementary material for: Radiolabeling of Angiostrongylus vasorum- and Crenosoma striatum larvae: a novel method using PET/CT to unveil larval migration in the gastropod intermediate host (Lissachatina fulica)
Source: Parasit Vectors. 2025 Nov 18;18:466. doi: 10.1186/s13071-025-07088-0 (PMC12625616; doi:10.1186/s13071-025-07088-0)
Supplement: Supplementary file 2 — Supplementary Material 2. Table S1 raw readings [file 13071_2025_7088_MOESM2_ESM.docx]

b

a


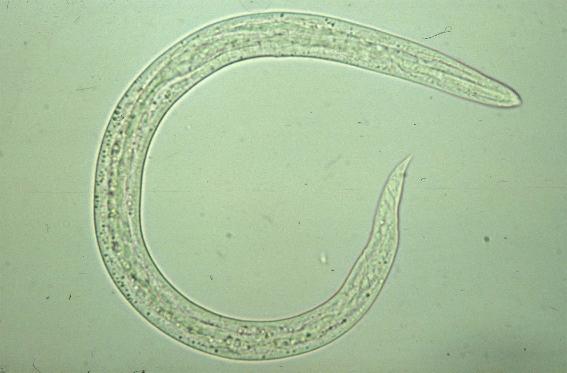

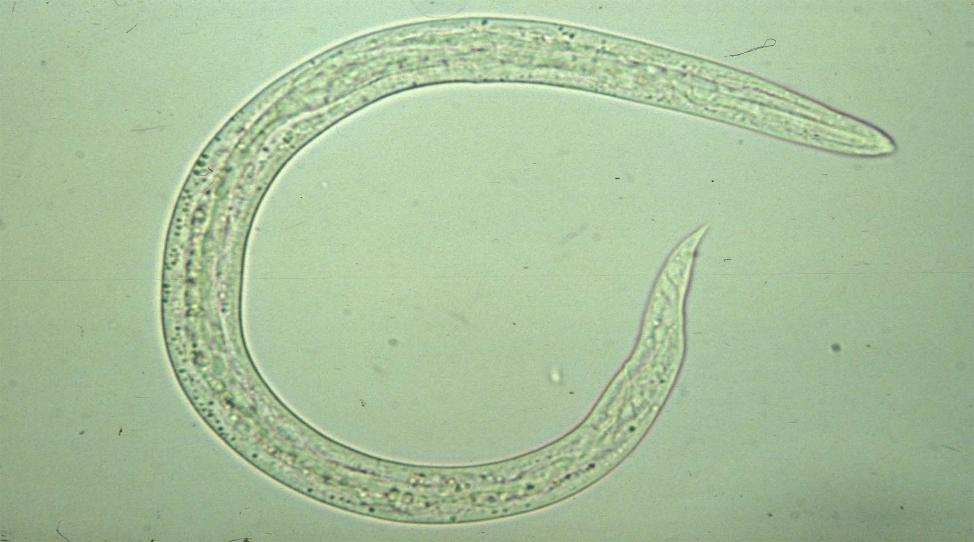

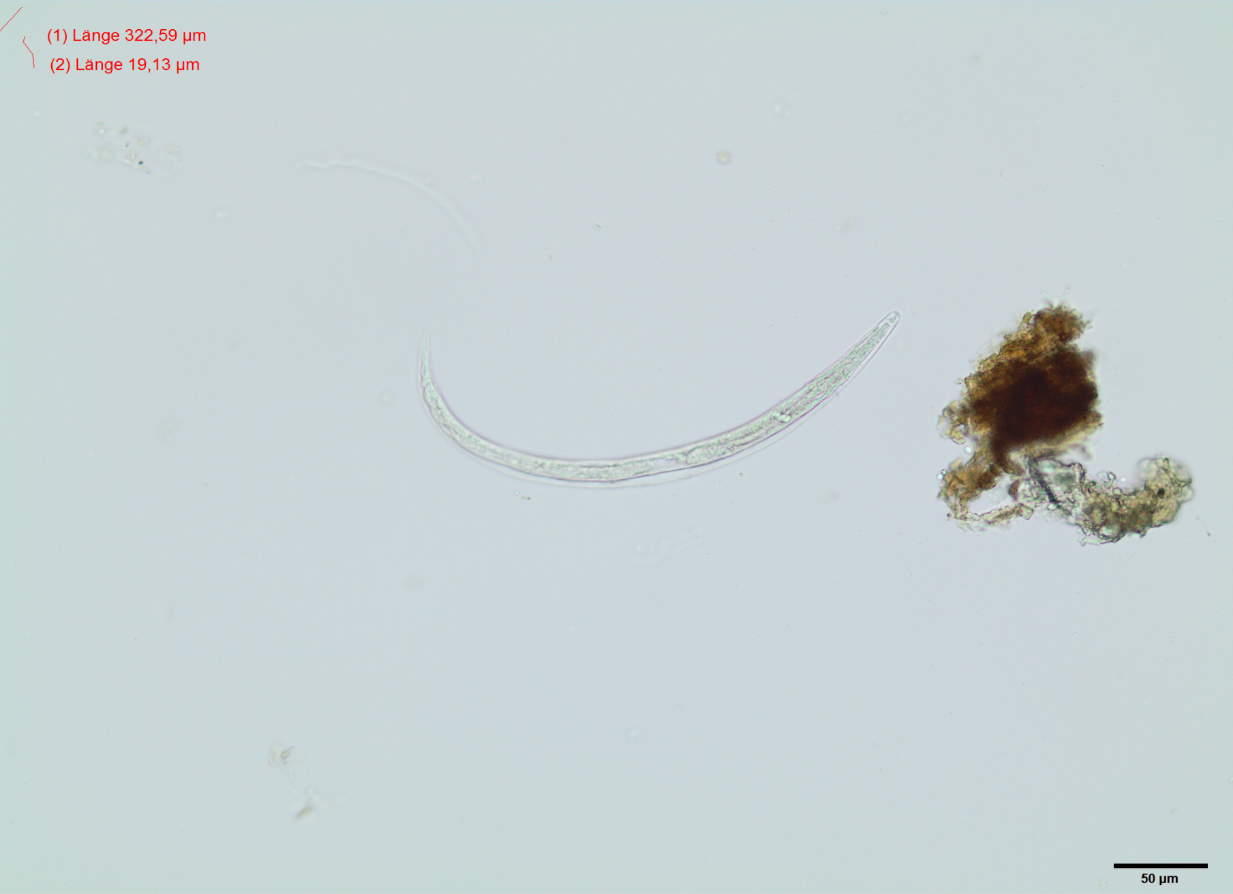

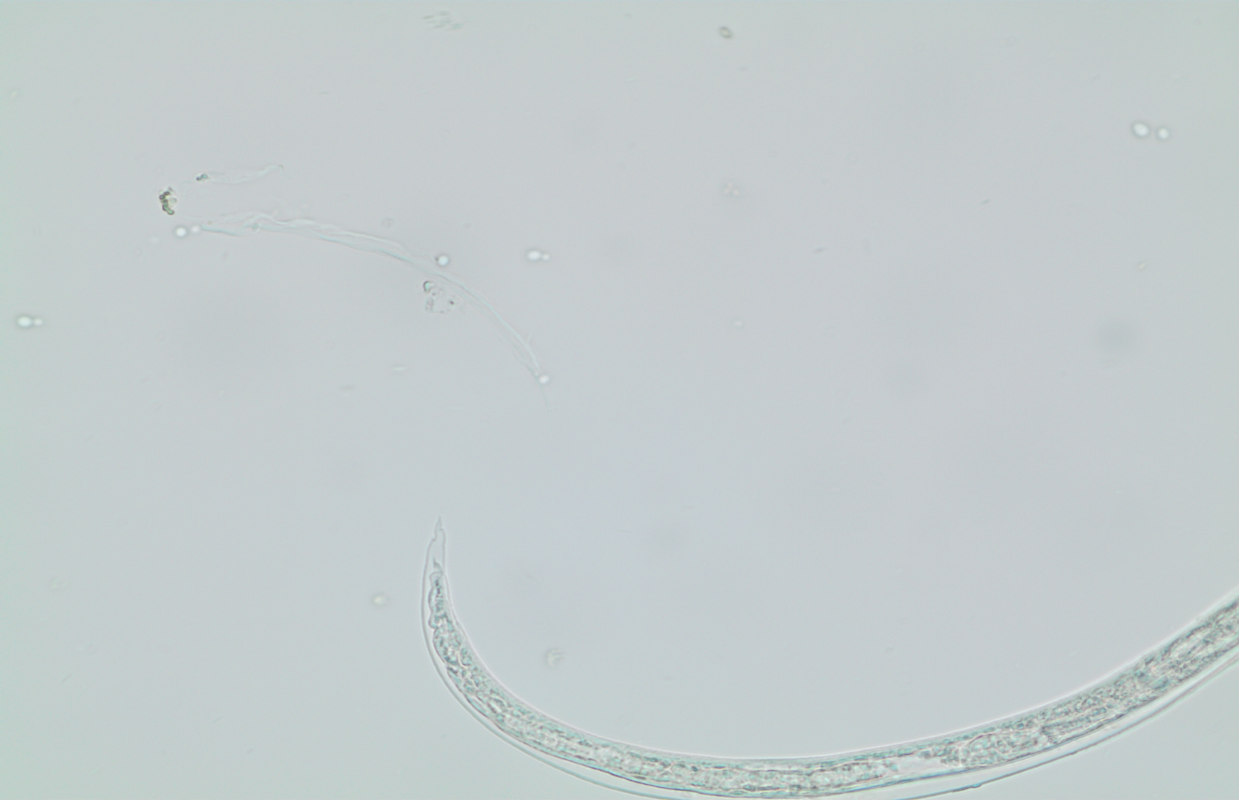


Supplementary figure 1. a: *Crenosoma striatum* first stage larva; b: *Angiostrongylus vasorum* first stage larva
